# A patient with left-sided inferior vena cava who received oblique lumbar interbody fusion surgery: a case report

**DOI:** 10.1186/s13256-020-2342-y

**Published:** 2020-01-27

**Authors:** Chen Liu, Jian Zhai, Quan Yuan, Yu Zhang, Hongguang Xu

**Affiliations:** 1 Spine Research Center of Wannan Medical College, Wuhu, 241001 Anhui China; 2 Department of Orthopaedics, Yijishan Hospital of Wannan Medical College, No. 2 Zheshan West Road, Wuhu, 241001 Anhui China; 3 Department of Radiology, Yijishan Hospital of Wannan Medical College, No. 2 Zheshan West Road, Wuhu, 241001 Anhui China

**Keywords:** Oblique lateral interbody fusion, Abdominal CTA, Inferior vena cava, Abdominal aorta, Left lateral decubitus position

## Abstract

**Background:**

Oblique lateral interbody fusion surgery has become increasingly popular for lumbar degenerative diseases. The oblique corridor is between the psoas muscle and the retroperitoneal vessels, and its use could result in decreased tissue trauma, minimal blood loss, and short operation times. Patients who undergo oblique lateral interbody fusion surgery are always placed in the right lateral position to avoid damage to the inferior vena cava, which is typically a right-sided vessel. There is a substantial risk of vascular injury during the operation if there are anatomical variations in the vessels.

**Case presentation:**

A 77-year-old man, of the Han nationality, with lumbar spinal stenosis underwent stand-alone oblique lateral interbody fusion surgery. Transverse magnetic resonance imaging of the lumbar spine indicated that his inferior vena cava was left-sided. A three-dimensional reconstructed image of abdominal computed tomography angiography showed that the inferior vena cava was located on the left side. Finally, the surgeon decided to change the position of our patient from a right lateral position to a left lateral position before the surgery.

**Conclusions:**

To date, this is the first reported case where a patient underwent oblique lateral interbody fusion surgery in a left lateral decubitus position due to a left-sided inferior vena cava. This case demonstrates that carefully reading radiological results is important for operation planning and avoiding anatomical complications.

## Background

Oblique lateral interbody fusion (OLIF) was first introduced for degenerative disc disease with minimal surgical trauma in 2012 [1]. Compared to previous fusion techniques, such as posterior lumbar interbody fusion (PLIF), anterior lumbar interbody fusion (ALIF), and extreme lateral interbody fusion (XLIF)/direct lateral interbody fusion (DLIF), OLIF is a direct mini-open approach via the psoas major muscle and abdominal aorta; for this surgery, it was decided that almost all patients would be placed in the right lateral decubitus position because the inferior vena cava (IVC) is typically a right-sided vessel. However, we report the first case of an adult with a left-sided IVC who underwent OLIF surgery with the use of a right abdominal oblique incision.

## Case presentation

A 77-year-old man of the Han nationality complained of numbness of his lower limbs for more than 5 years. In addition, his walking distance was less than 100 meters. A neurological examination suggested tenderness in the area of the lumbosacral spinous processes and bilateral paravertebral muscle. The feeling of numbness on the superficial skin of his bilateral lower limbs decreased. His Oswestry Disability Index (ODI) score was 24 and his Visual Analogue Scale (VAS) score was 5 before the operation. He had no history of hypertension, diabetes, or surgery. He also had no tobacco smoking or alcohol history. He took celecoxib orally, twice a day, one tablet a time; his pain was obviously not relieved. Before the operation, the white blood cell of our patient was 5.5 × 10^9^/L, the red blood cell was 4.26 × 10^12^/L, the hemoglobin (HGB) was 129 g/L, and the platelets (PLT) was 170 × 10^9^/L. As for his liver and renal functions, his alanine aminotransferase (ALT) was 11 U/L and his aspartate aminotransferase (AST) was 2211 U/L. His urea was 6.07 mmol/L and his creatinine was 71.9 umol/L. The tests of urine analysis, serology, and microbiology were normal.

**Fig. 1:**
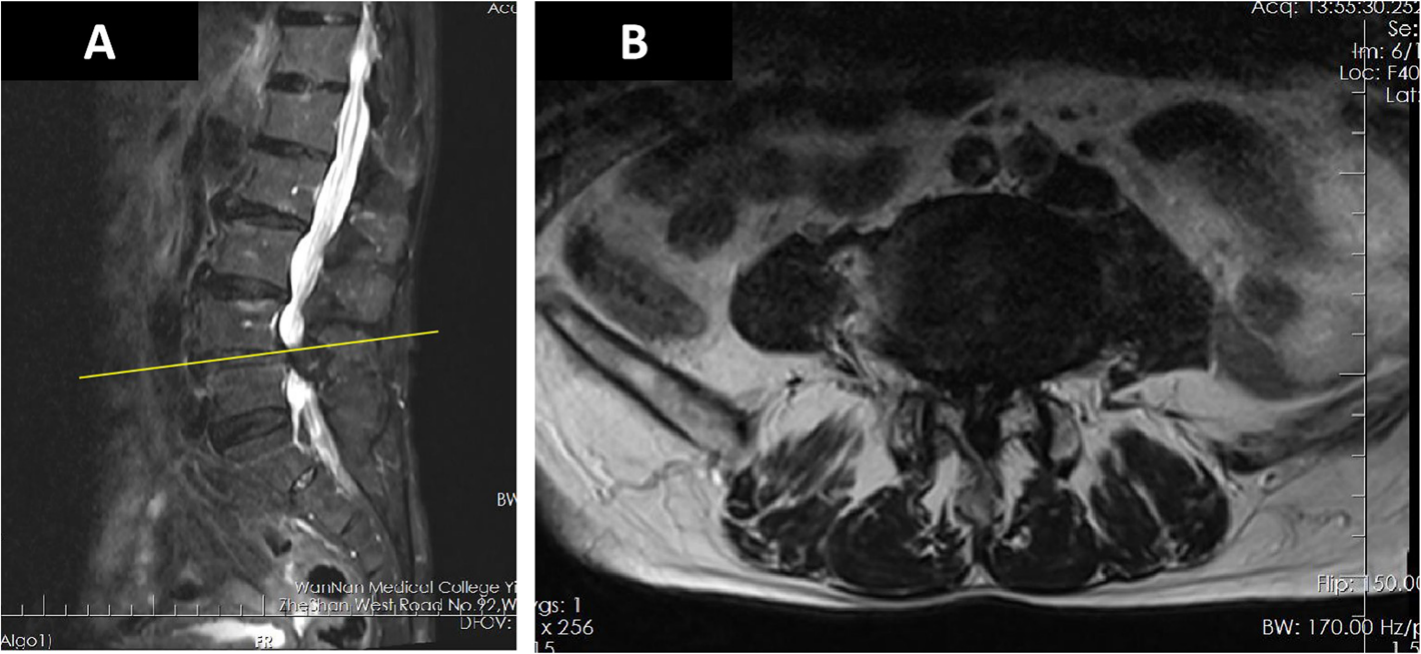
Sagittal (**a**) and transverse (**b**) fat-suppressed magnetic resonance imaging of the lumbar spine indicated that the spinal canal was extremely narrow

**Fig. 2:**
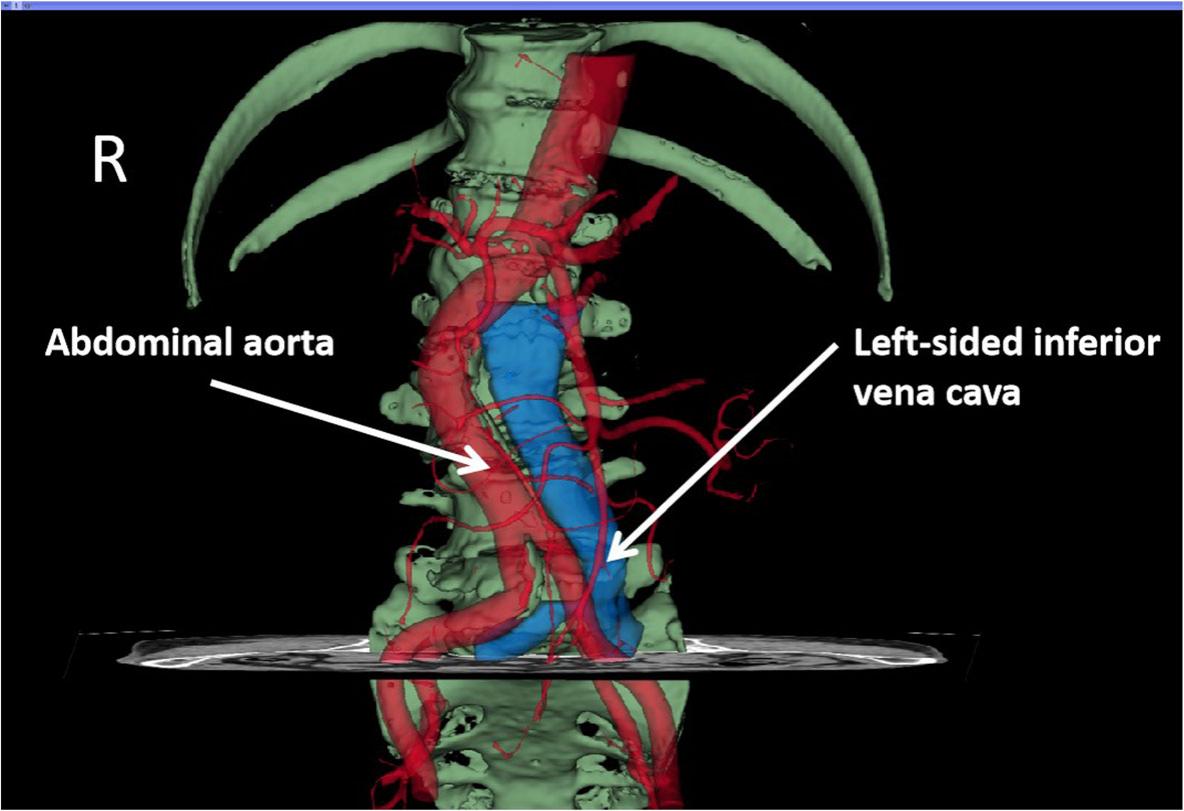
A reconstruction computed tomography angiography of retroperitoneal vessels and lumbar vertebrae indicated that the inferior vena cava was located on the left side

**Fig. 3:**
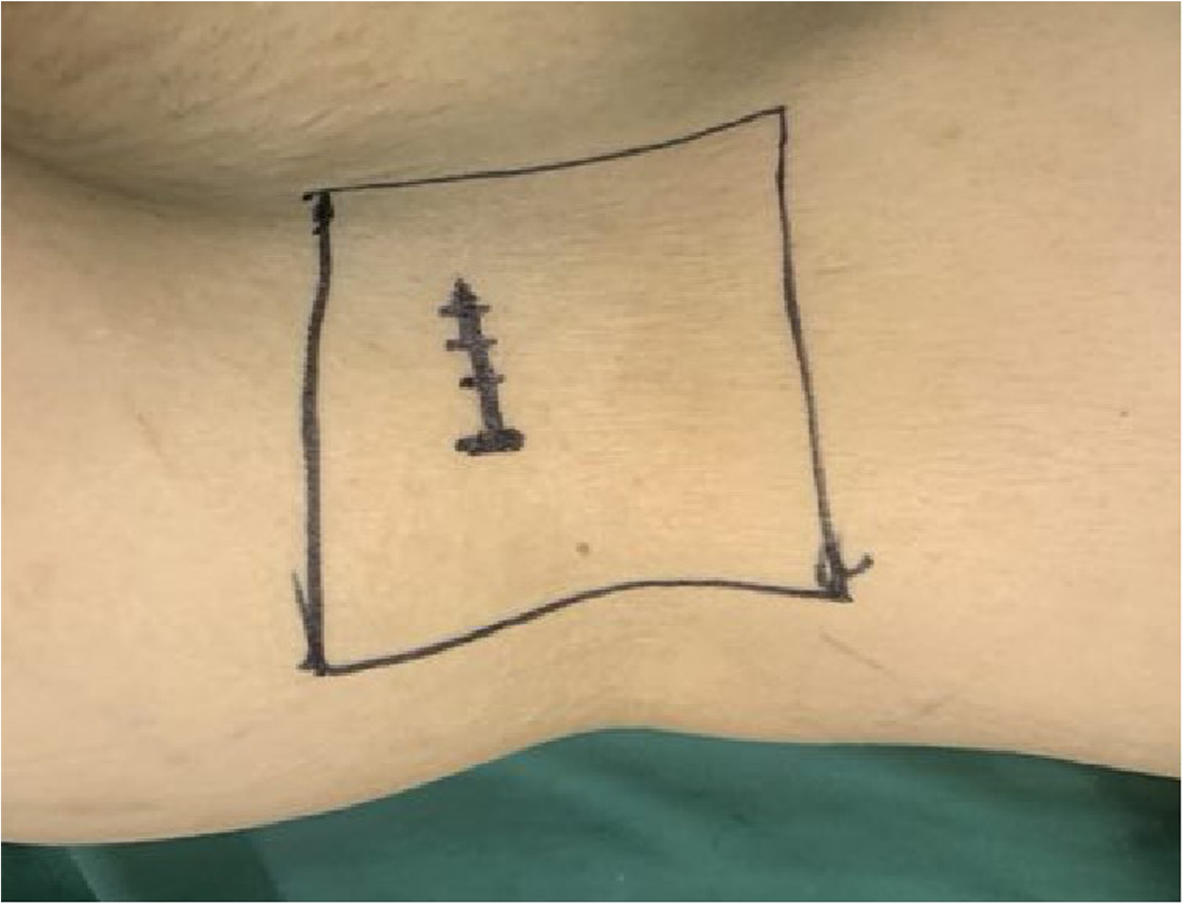
A transverse incision was marked on the skin before the oblique lateral interbody fusion surgery

Lumbar magnetic resonance imaging (MRI) (Fig. 1) indicated that the spinal canal of L4/5 was extremely narrow. He was diagnosed as having spondylolisthesis and lumbar spinal stenosis and was ready for stand-alone OLIF surgery in terms of clinical manifestations and imaging examinations. Unlike most other people, in this case, his IVC was discovered to be left-sided from the MRI image. In addition, we confirmed that he had a left-sided IVC with reconstruction computed tomography angiography (CTA) of the retroperitoneal vessels and lumbar vertebrae (Fig. 2). Finally, he underwent L4/5 stand-alone OLIF surgery under general anesthesia in the left lateral decubitus position. In the left lateral position, a transverse skin incision of 5 cm was made at the area of the right side of his abdomen, which was in the same horizontal plane as the L4/5 intervertebral disc (Fig. 3). His abdominal wall muscles were bluntly separated. The retroperitoneum was entered via blunt separation with the fingers; then, the psoas muscle was retracted posteriorly, and the abdominal vessels were retracted anteriorly. A guidewire was inserted in the middle of the target intervertebral disc with the help of a C-arm. Sequential dilators were placed over the guidewire; then, a lighted retractor was placed over the dilators and fixed to the vertebral body with a pin, and the operation field was exposed. The annulus fibrosus and the nucleus pulposus were removed with a nucleus pulposus clamp. Then, the cartilage endplates were resected for exposure of the bony endplates. A wide and lordotic intervertebral fusion cage of 14 × 55 × 6 mm (Medtronic Clydesdale, Memphis, Tennessee) packed with allograft bone was inserted into the L4/5 disc with the guidance of the C-arm. Then, the incision was closed layer by layer. The blood loss was approximately 30 ml.

**Fig. 4:**
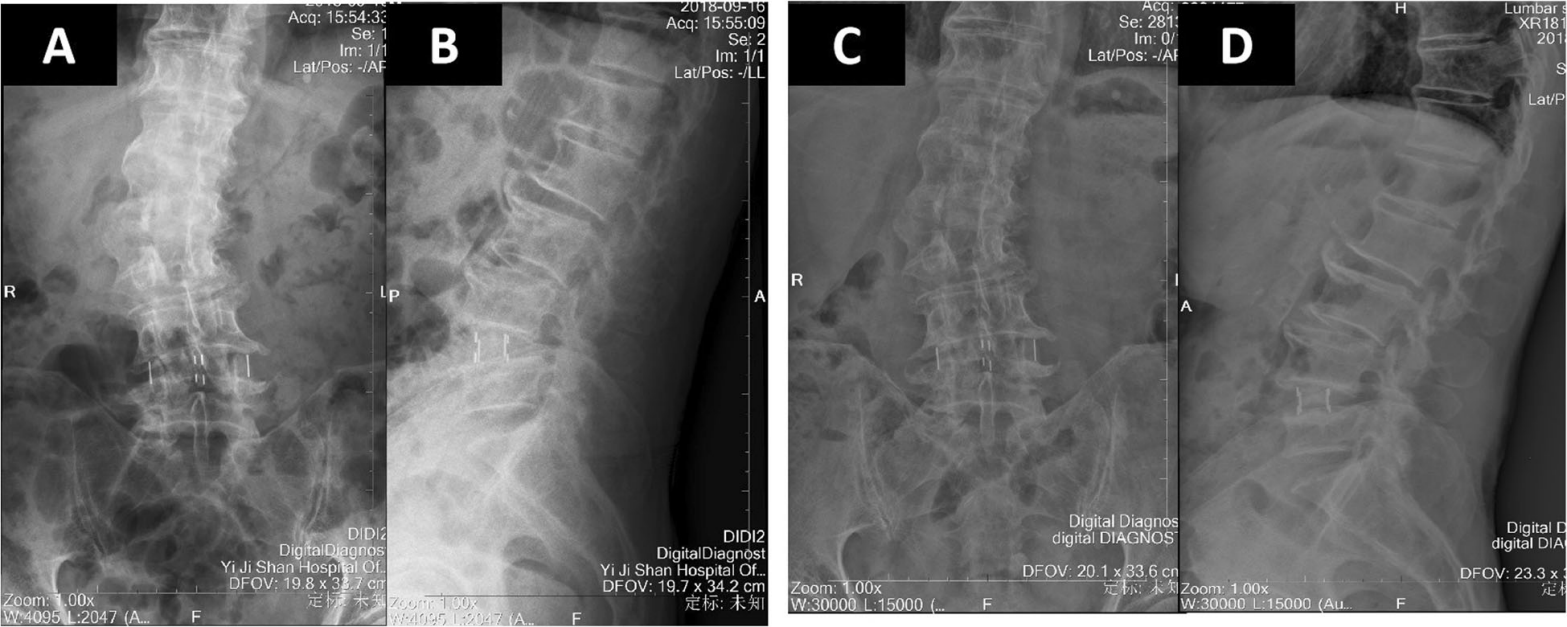
X-ray of the lumbar spine after the stand-alone oblique lateral interbody fusion operation (**a**, **b**) and three-month follow-up (**c**, **d**)

Our patient began to practice walking the day after the operation with waist protection, and his symptoms improved. A lumbar X-ray (Fig. 4a, b) after the surgery showed that the cage was located in the center, and he was discharged from our hospital 2 days after the surgery. After 3 months, the symptoms of lumbar and leg pain were significantly alleviated, and his ODI and VAS scores were 20 and 3, respectively. A lumbar X-ray also indicated that the location of the cage was perfect (Fig. 4c, d). Half a year after the surgery, our patient did not complain of low back and leg pain. In addition, his ODI score was 19 and his VAS score was 2.

## Discussion

At present, there are a number of studies on OLIF for the treatment of lumbar degenerative diseases, such as spondylolisthesis, lumbar spinal stenosis, and spinal deformity [2, 3]. Although OLIF reduces the risks of direct damage to the dural sac and the nerve root compared with traditional PLIF or transforaminal lumbar interbody fusion (TLIF), OLIF is associated with catastrophic complications, such as large retroperitoneal vessels, due to the limited operation approach between the psoas muscle and retroperitoneal vessels. It is very important for surgeons to have a good understanding of the anatomical relationship between the retroperitoneal vessels and the vertebral bodies to minimize the risks for these complications. The oblique access corridor has been investigated by many researchers. Liu *et al*. [4] selected imaging data from 60 adults who underwent abdominal CTA and T12–S1 vertebral computed tomography (CT) with three-dimensional reconstruction, and the vascular window, bare window, and psoas major window were measured. Davis *et al*. [5] dissected 20 fresh-frozen full-torso cadaveric specimens and examined the oblique anatomical corridor of the L2–S1 discs. All measurements of the anatomical corridor were defined as the left lateral border of the aorta (or iliac artery). Almost all patients were placed in the right lateral position to receive the operation. This was the first report of using the left lateral position due to a left-sided IVC.

Variations in the location of the IVC are common. To date, there have been more than 60 kinds of congenital malformations of the IVC, including duplication, interruption, and transposition anomalies. Dual IVC is the most common variant of IVC anomaly. It results from the persistence of both supracardinal veins. The incidence of duplicated IVC accounts for 0.2–0.5% in the general population [6, 7]. Other variations include a lack of a hepatic segment of the IVC from the azygos vein, a left renal vein from the circumferential aorta, complete absence of the IVC, and so on. Among the various kinds of anatomical variations, a left-sided IVC is extremely rare. Ang *et al*. [8] investigated published studies of left-sided IVC between 2000 and 2011 and found that the prevalence of a left-sided IVC ranged from 0.1% to 0.4%. The embryonic development of the IVC is complex. The IVC is formed by the successive development and degeneration of the posterior main vein, the inferior main vein, and the superior main vein. During this process, any abnormal development or normal degeneration can lead to different forms of IVC malformations. For the diagnosis of a left-sided IVC, digital subtraction angiography (DSA) can accurately display the whole lesion and measure the pressure of the right atrium and the IVC. DSA can provide a reliable basis for positioning, qualitative diagnosis, and guiding interventional therapy. The course of the IVC and its anatomical relationship with the abdominal aorta can also be well displayed by MRI and CT, especially with enhanced scans, to make a definitive diagnosis. For left-sided IVC malformations, there are no clinical symptoms or serious consequences. However, surgeons should pay attention to the operation of the related areas, and careful preoperative consideration should be taken to prevent intraoperative injuries.

**Fig. 5:**
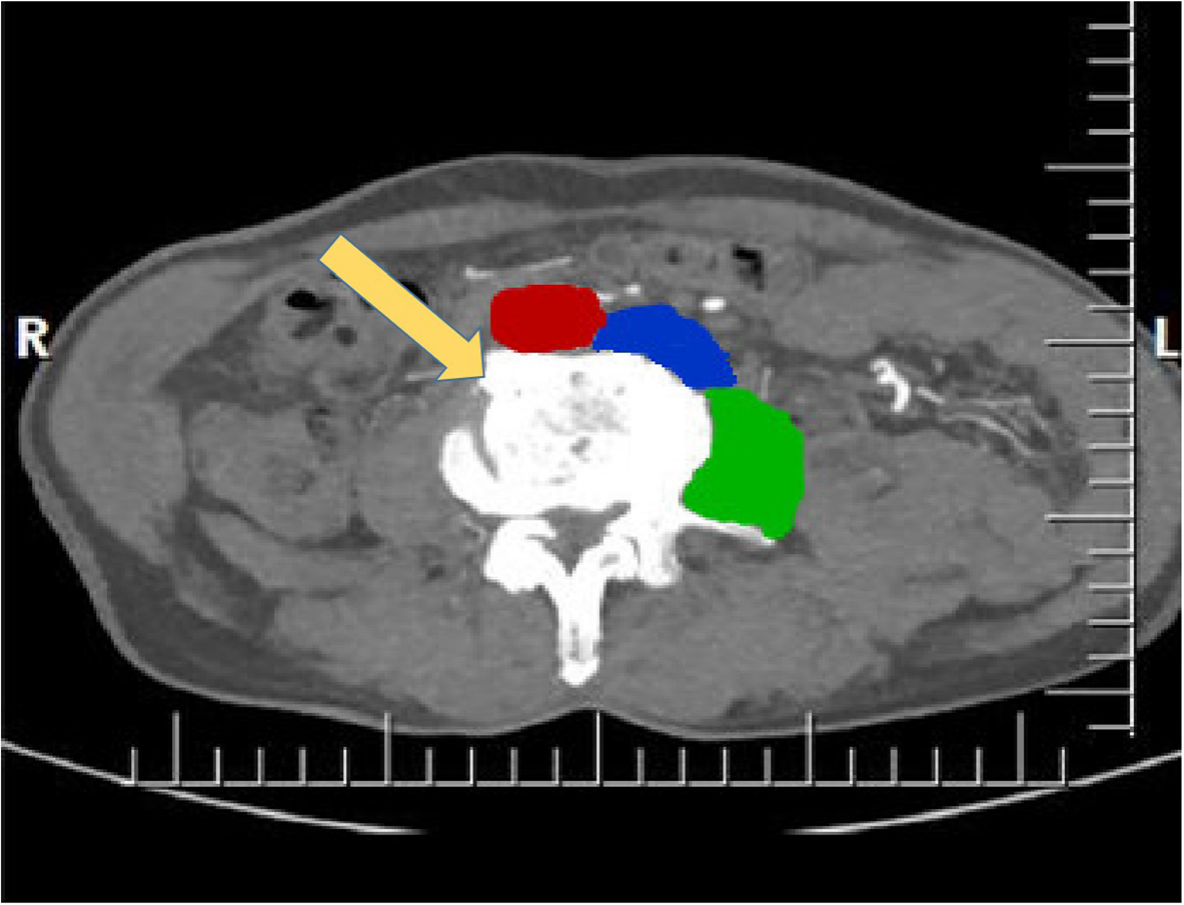
A transverse image of a contrast-enhanced abdominal computed tomography scan. The *red region* refers to the abdominal aorta; the *blue region* represents the inferior vena cava; and the *green region* represents the psoas major muscle. The *yellow arrow* represents the operation window

In this case, the left-sided IVC was located between the abdominal aorta and the left psoas major muscle at the level of the L4/5 intervertebral disc, as shown in Fig. 5. Taking the possible vascular injury into consideration, we successfully completed the OLIF surgery through a right operative window to avoid damaging the variant IVC.

## Conclusions

In fact, a left-sided IVC is uncommon but clinically significant. Up to now, this is the first reported case of a patient who was diagnosed as having lumbar spinal stenosis and underwent stand-alone OLIF surgery in the left lateral decubitus position due to the presence of a left-sided IVC. Although we did not consult the vascular service in this case, it is recommended that they should be involved when there are major vascular anomalies present. OLIF can be possible from both sides by an expert surgeon as there are no major technical challenges; however, vascular anomalies and variations must be kept in mind and radiologically identified. Awareness of variations in the IVC is crucial for the choice of an operation window during OLIF surgery, which could avoid many anatomically relevant complications. This patient may offer a good example of anatomical variations; surgeons should be aware of anatomical variations in vessels before performing operations.

## Data Availability

Not applicable.

## Abbreviations

ALIF: Anterior lumbar interbody fusion
ALT: Alanine aminotransferase
AST: Aspartate aminotransferase
CT: Computed tomography
CTA: Computed tomography angiography
DLIF: Direct lateral interbody fusion
DSA: Digital subtraction angiography
HGB: Hemoglobin
IVC: Inferior vena cava
MRI: Magnetic resonance imaging
ODI: Oswestry Disability Index
OLIF: Oblique lateral interbody fusion
PLIF: Posterior lumbar interbody fusion
PLT: Platelets
TLIF: Transforaminal lumbar interbody fusion
VAS: Visual Analogue Scale
XLIF: Extreme lateral interbody fusion
